# Do Genetic Polymorphisms Modulate Response Rate and Toxicity of Cisplatin Associated With Radiotherapy in Laryngeal Squamous Cell Carcinoma? A Case Report

**DOI:** 10.1097/MD.0000000000000578

**Published:** 2015-04-24

**Authors:** Leisa Lopes-Aguiar, Marília Berlofa Visacri, Carolina Marques Lopes Nourani, Ericka Francislaine Dias Costa, Guilherme Augusto Silva Nogueira, Tathiane Regine Penna Lima, Eder Carvalho Pincinato, Patrícia Moriel, João Maurício Carrasco Altemani, Carmen Silvia Passos Lima

**Affiliations:** From the Department of Internal Medicine (LLA, CMLN, EFDC, GASN, TRPL, ECP, CSPL); Department of Clinical Pathology Brazil (MBV, PM); and Department of Radiology (JMCA), Faculty of Medical Sciences, University of Campinas, Campinas, São Paulo, Brazil.

## Abstract

Cisplatin (CDDP) plus radiotherapy (RT) has been used to treat advanced laryngeal squamous cell carcinoma (LSCC) patients. Single nucleotide polymorphisms (SNPs) may be responsible for differences in chemo/radiosensitivity and side effects in those patients. We reported an advanced LSCC patient, who obtained durable complete response and unexpected pronounced toxicity during CDDP and RT, possibly due to SNPs in genes that modulate the effects of this therapeutic modality. Case presentation: A 30-year-old man with advanced LSCC obtained durable complete response and severe alopecia and pancytopenia after standard and reduced doses of CDDP and RT. Analyses of SNPs revealed that the patient presented *GSTT1* deletion, variant *MSH3* 1045ThrThr, wild *GSTP1* 105IleIle, and wild *BAX* -248GG genotypes, which were previously described in association with abnormal detoxification, DNA repair, and damaged cell apoptosis, respectively. Seven other advanced LSCC patients with *GSTT1* gene, *MSH3* AlaAla or AlaThr, *GSTP1* IleVal or ValVal, and *BAX* GA or AA genotypes served as controls of the study. Only 1 control presented complete response; the other 6 controls obtained partial response of short duration. Four and 3 controls presented grade 1 or 2 and grade 3 anemia or leukopenia during treatment, respectively. The CDDP level in urine collected after CDDP infusion in the reported patient was lower than the median value obtained in controls, suggesting a higher amount of intracellular CDDP in the reported case.

The data suggest, for the first time, that inherited abnormalities in intracellular detoxification of CDDP, DNA repair of lesions induced by CDDP and RT, and damaged cell apoptosis may alter treatment response and toxicity in LSCC, but should be confirmed by large pharmacogenomic studies.

## INTRODUCTION

Cisplatin (CDDP) plus radiotherapy (RT) has been used for organ preservation in advanced laryngeal squamous cell carcinoma (LSCC).^1^

Complete and partial responses were identified in approximately 90% and 10% of patients with stage III or IV LSCC treated with CDDP and RT, respectively, which persisted for 2 years in about 80% of cases.^1^ Grade 3 or 4 anemia, leucopenia, or thrombocytopenia attributed to treatment was seen in 47% of treated patients.^1^ To the best of our knowledge, there are no descriptions in the literature regarding pancytopenia after standard doses of CDDP, and alopecia is a rare event in treated patients.^2^

CDDP develops covalent bifunctional DNA adducts with cellular DNA. CDDP-DNA adducts are repaired particularly by nucleotide excision pathways, and when the repair of DNA lesions are not possible, apoptosis is activated, leading damaged cells to death. Similarly, ionizing radiation induces damage to DNA directly by action of photons and/or indirectly by liberation of free radicals, which induces damaged cell apoptosis when not adequately repaired, particularly by mismatch and base excision pathways.

Variations in tumor sensitivity to CDDP and RT, as well as side effects of therapeutic modalities have been attributed to distinct activities of proteins, encoded by single nucleotide polymorphisms (SNPs) in different genes. The main SNPs potentially involved in modulation of effects of this treatment are presented in Table 1.

**TABLE 1:**
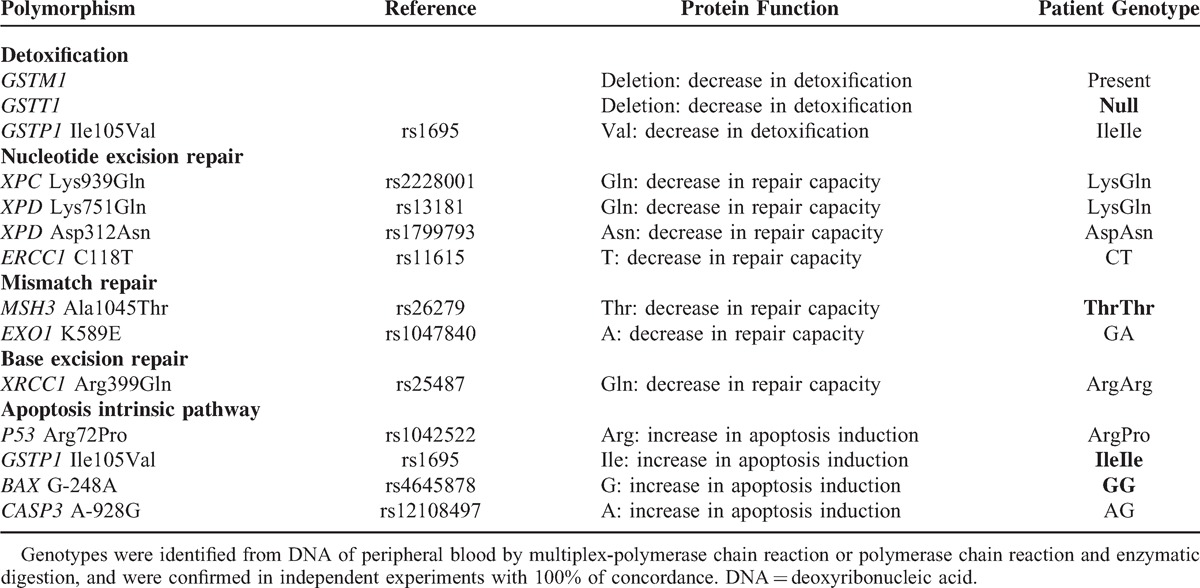
Polymorphisms Involved in Detoxification of Cisplatin and Mechanisms of DNA Repair and Apoptosis Pathway

*GSTM1*^3^ and *GSTT1*^4^ genotypes, enrolled in detoxification, *XPD* Lys751Gln and Asp312Asn,^3^*MSH3* Ala1045Thr,^5^*XRCC1* Arg399Gln^3,6,7^ SNPs, enrolled in DNA repair, and *P53* Arg72Pro,^3^ and *GSTP1* Ile105Val^3,8^ SNPs, enrolled in damaged cell apoptosis, were associated with variable response, toxicity, progression-free survival, and overall survival of patients with different tumors treated with platinum-based chemotherapy with or without RT; however, only few studies were conducted in head and neck SCC (HNSCC) patients.^4,6–8^

We report a man with advanced LSCC, with concurrent inherited abnormalities in CDDP and RT metabolism, which seemed to confer sensitivity to treatment even though with pronounced side effects.

## CASE REPORT

A 30-year-old man, drinker and smoker, was seen as an outpatient at the University Hospital in October 2011 with hoarseness for the past 4 months. A vegetative lesion occupying anterior 2/3 of right vocal cord and anterior third of left vocal cord was detected during laryngoscopy. Computed tomography of neck evidenced uptake vegetating lesion in the right vocal cord and bilateral lymph node metastasis (level 2) (15.0 and 23.2 mm). After histological evaluation of tumor fragment, the diagnosis of moderately differentiated LSCC was established. The tumor was staged as IVa (T2N2cM0) according to the American Joint Committee on Cancer Staging criteria. The human papillomavirus type 16 was negative in tumor.

**FIGURE 1:**
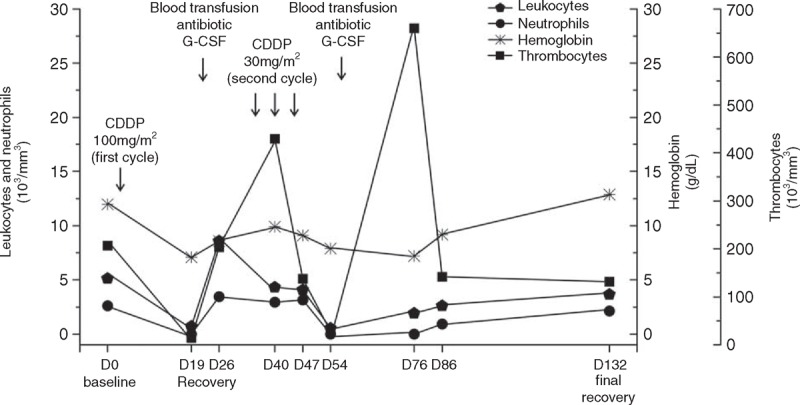
Leukocytes, neutrophils, hemoglobin, and thrombocytes values before treatment (baseline on day 0) and after treatment with cisplatin-based chemotherapy (first cycle dose: 100 mg/m^2^ and second cycle dose: 30 mg/m^2^ during 3-week applications) concomitant with radiotherapy. During the first cycle, on day 19, and during the second cycle, on day 54, severe pancytopenia occurred and required blood transfusion and antibiotic, as well as the administration of granulocyte-colony-stimulating factor (G-CSF). Hematologic recovery occurred on day 26 (after the first cycle of CDDP) and final recovery occurred on day 132 (after the second cycle of CDDP). CDDP = cisplatin.

The patient entered into a treatment protocol consisting of “in bolus” intravenous CDDP at dose of 100 mg/m^2^ on days 1, 22, and 43, and concurrent single daily fractionated radiation (2 Gy/day). As antiemetic protocol, he received intravenous 24 mg ondansetron and 20 mg dexamethasone, before CDDP infusion, as well as oral 8 mg dexamethasone (every 12 hours) and 10 mg metoclopramida (every 6 hours) during 3 days after each CDDP infusion. On day 19, after developing grade 2 alopecia, fever, severe pancytopenia (leukocytes 0.7 × 10^3^/mm^3^, neutrophils 0.0 × 10^3^/mm^3^, hemoglobin 7.8 g/dL, thrombocytes 14.0 × 10^3^/mm^3^), the patient received blood transfusion, intravenous 500 mg vancomycin (every 6 hours), 1 g cefepime (every 8 hours), and subcutaneous 300 μg of granulocyte-colony-stimulating factor (once a day). After hematologic recovery, intravenous CDDP 30 mg/m^2^ was administered on days 33, 40, and 47. On day 54, the patient developed another episode of fever, pancytopenia (leukocytes 0.5 × 10^3^/mm^3^, neutrophils 0.0 × 10^3^/mm^3^, hemoglobin 7.9 g/dL, thrombocytes 28.0 × 10^3^/mm^3^), requiring supportive treatment. After this episode, the patient received exclusive RT. Hematologic recovery was seen only on day 132 (Figure 1).

The computed tomography of neck evidenced complete remission of LSCC in April 2012, according to the Response Evaluation Criteria in Solid Tumors, which was maintained until the last follow-up in January 2015 (34 months of duration).

The patient presented *GSTT1* deletion, variant *MSH3* ThrThr genotype, and wild *GSTP1* IleIle and *BAX* GG genotypes (Table 1).

All LSCC outpatients with clinical and tumor aspects similar to those seen in the reported case, and with *GSTT1* gene, *MSH3* AlaAla or AlaThr, *GSTP*1 IleVal or ValVal, and *BAX* GA or AA genotypes seen in our service during the last 3 years (2012–2014) served as controls of the study (n = 7). All controls were treated with intravenous CDDP (100 mg/m^2^ on days 1, 22, and 43) and concurrent single daily fractionated radiation (2 Gy/day). Only 1 control presented complete response; the remaining 6 controls obtained partial response of short duration. Four and 3 controls presented grade 1 or 2 and grade 3 anemia or leucopenia analyzed by the National Cancer Institute criteria during treatment, respectively. Alopecia was not seen in any control of the study. Surgical resection of residual tumors was not performed due to locoregional irresistibility or patient refusal. At the end of the study, 5 out of 7 controls died due to tumor effects. The CDDP level was measured by high-performance liquid chromatography in 12-hour urine collected of the reported case and controls after the first intravenous administration of CDDP. The CDDP level in urine of the reported patient (2.20 mg/m^2^) was lower than the median level of CDDP found in urine of controls (9.90 mg/m^2^; variation: 3.67–26.00; *P* = 0.01, 1-sample exact Wilcoxon signed rank test) (Table 2).

**TABLE 2:**
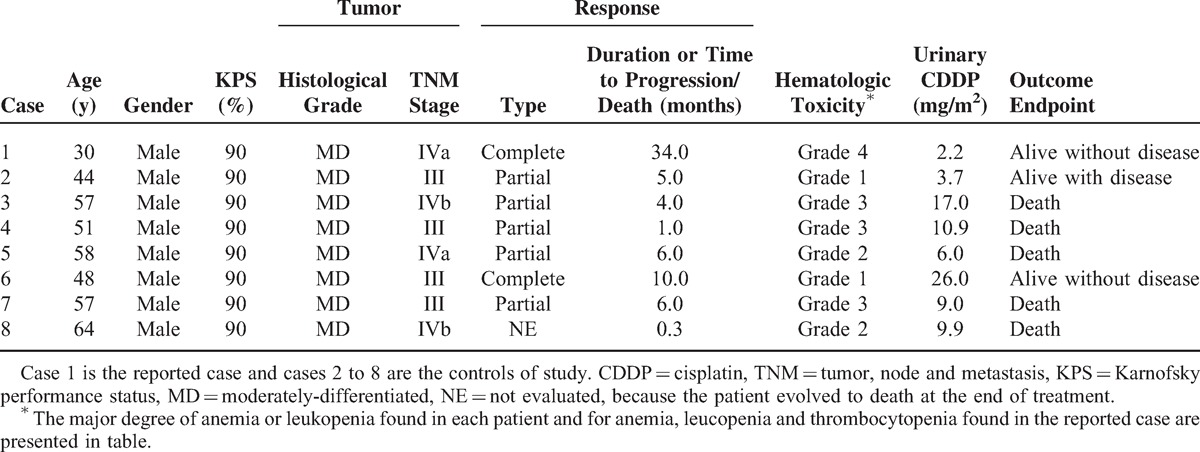
Clinical Features, Tumoral Aspects, and Urinary Cisplatin Excretion in Patients With Laryngeal Squamous Cell Carcinoma

## DISCUSSION

In this case report, an outpatient with advanced LSCC treated with CDDP and RT presented durable complete response, and unexpected severe pancytopenia and alopecia. In fact, CDDP and RT may determine complete response in HNSCC patients, and it may be even longer than 2 years.^1^ However, alopecia grade 2 is a rare event,^2^ and severe pancytopenia was reported only in cancer patients treated with CDDP overdose.^9^ To the best of our knowledge, there are no descriptions in the literature regarding pancytopenia after standard doses of CDDP.

Thus, we checked whether inherited abnormalities in intracellular detoxification of CDDP, DNA repair, and damaged cell apoptosis could constitute a plausible explanation for the uncommon findings seen in the reported patient. SNP analyses revealed that the patient presented deletion of *GSTT1*, associated with decreased detoxification of CDDP and RT products,^3^ variant *MSH3* ThrThr genotype, associated with possible decrease in DNA repair by mismatch pathaway,^10^ and wild *GSTP1* IleIle^3,8^ and *BAX* GG genotype, associated with increase in apoptosis induction by intrinsic pathway.^11^

No association between homozygous deletion of *GSTT1* and response rate in HNSCC,^4^ but radiosensitivity associated with *MSH3* Ala allele in breast cancer patients,^5^ was previously described during treatment using CDDP-based chemotherapy and RT. The wild IleIle genotype of *GSTP1* Ile105Val SNP conferred higher response and longer survival to ovarian cancer patients treated with CDDP-based chemotherapy,^3^ but not to HNSCC patients treated with RT and CDDP.^8^ Severe neutropenia was more frequent in ovarian cancer patients with AspAsn and ArgArg genotypes of *XPD* Asp312Asn and *XRCC1* Arg399Gln SNPs, respectively, who were treated with standard dose of CDDP.^3^ HNSCC patients with Gln allele of *XRCC1* Arg399Gln SNP were under high risk of mucositis if treated with RT.^7^ Moreover, variant ThrThr genotype of *MSH3* Ala1045Thr SNP was associated with severe radiation dermatitis in breast cancer patients.^5^

Previous descriptions, even incipient, indicate that SNPs with activity in intracellular detoxification, DNA repair, and/or apoptosis of damage cells may alter response and toxicity to therapy, and prognosis of cancer patients treated with CDDP-based chemotherapy and RT. Facing these descriptions and our findings, we hypothesized that homozygous deletion of *GSTT1* gene decreased intracellular detoxification of CDDP,^12^ variant Thr allele of *MSH3* Ala1045Thr SNP decreased DNA repair by mismatch pathway^13^ and wild Ile^14^ and G^11^ alleles of *GSTP1* Ile105Val and *BAX* G-248A SNPs, respectively, increased apoptosis of tumor and normal cells by intrinsic pathway, determining durable complete response, pronounced pancytopenia, and total alopecia in the reported case, while the remaining genotypes of the genes had the opposite effects on the other 7 patients enrolled in the study.

We also found in study that CDDP level in urine of the reported case was lower than levels found in urine of controls. It is already well known that pharmacokinetic studies of CDDP are usually carried out in blood samples collected between 1 and 18 hours after CDDP infusion.^15,16^ However, since outpatients were enrolled in this study, only 12-hour urine was available for CDDP analysis. Lanjwani et al (2006)^16^ found a good correlation between levels of CDDP in blood and urine samples, and postulated that both methods may be used in pharmacokinetic studies of CDDP. Thus, we believe that urinary CDDP levels found in our study might reflect the plasma concentration of the agent in our patients. Conversely, correlation between CDDP concentration in plasma and formation of CDDP-DNA adducts in leukocytes of patients with cancer is controversial.^17,18^ Since CDDP urinary concentration may reflect CDDP plasma levels, and CDDP plasma concentration may be correlated with CDDP-DNA adducts in cells, we hypothesized that low urinary CDDP level in urine of the reported case might be associated with retention of CDDP in normal and tumor cells in our patients, with formation of CDDP-DNA adducts and consequent good response to therapy and pronounced toxicity, while high concentration of CDDP in urine of controls might be correlated with opposite findings.

Our data suggest, for the first time, that homozygous *GSTT1* deletion, *MSH3* Ala1045Thr, *GSTP1* Ile105Val, and *BAX* G-248A SNPs can act together and modulate the response rate and toxicity of CDDP associated with RT in the patient with LSCC. However, we believe that large studies in pharmacogenomics are required to clearly define the roles of the above-mentioned SNPs on modulation of CDDP and RT effects in those patients.
